# The Influence of Manipulating and Accentuating Task-Irrelevant Information on Learning Efficiency: Insights for Cognitive Load Theory

**DOI:** 10.5334/joc.361

**Published:** 2024-04-18

**Authors:** Batel Hazan-Liran, Paul Miller

**Affiliations:** 1 Tel-Hai Academic College, IL; 2 University of Haifa, IL

**Keywords:** Learning, Working Memory, Cognitive Load Theory, Stroop Effect, Extraneous Cognitive Load

## Abstract

The paper endorses Cognitive Load Theory and offers insights into the characterization of the mechanisms underlying extraneous cognitive load and their impact on basic learning. Students were asked to learn associations between eight base-code words and eight digits, based on an example, and to rapidly apply their new knowledge in a test section. Two groups of 60 university students participated in two experiments. The study was implemented as two distinct experiments, one using color names (e.g., blue, yellow) and the other using color-related word concepts (e.g., sky, banana) for stimulation. Each experiment had two conditions that manipulated the location and salience of task-irrelevant color information (extraneous cognitive load) and its congruity with the digits’ corresponding base-code words. Findings indicated extraneous cognitive load has the potential to both sustain and undermine learning processes by varying the overall cognitive load, with gains and costs in learning efficiency resulting from essentially different processing scenarios.

## Introduction

Humans’ ability to learn is fundamentally interrelated with working memory (WM) and prior knowledge and experience stored in long-term memory, including the way these are recruited to process the various demands arising from to-be-learned information (Baddeley, 1986, 2012). This complex cognitive architecture is conceptualized in Cognitive Load Theory’s (CLT) formulation of how individuals deal with the acquisition of new information (Sweller, 2011; Sweller et al., 2011). CLT’s basic assumption is that learning efficiency is inherently linked to two kinds of cognitive load: intrinsic and extraneous.

Intrinsic cognitive load is created by task-relevant information held in WM and used in the acquisition of new knowledge and/or the application of existing knowledge to tasks (Plass & Kalyuga, 2019; Sweller, 1994; Sweller & Chandler 1994). The extent of this load is principally determined by the number of elements a learner simultaneously has to process, i.e., element interactivity. Given the availability of numerous learned schemas stored in long-term memory, element interactivity can be reduced by merging interconnected information into broader knowledge units and by so doing, reduce cognitive load (Plass & Kalyuga, 2019). In other words, intrinsic cognitive load is considered the productive component of learning, with the level of productivity modified by the degree of element interactivity (Sweller et al., 1998; van Merriënboer & Sweller, 2005).

The overall cognitive load, beyond being determined by task-inherent factors (number of to-be-processed information units, availability of task-appropriate schemas, etc.), is often additionally altered by the entrance of task-irrelevant information into cognitive processes, as this information has an effect on materials held in WM (Sweller, 2010; Sweller et al., 2011; Sweller & Chandler, 1994; van Merriënboer & Ayres, 2005). This extraneous cognitive load has been claimed to have the ability to impact learning outcomes negatively because of a scarcity of WM capacity, particularly when learning involves the processing of high-element-interactivity materials (Lavie et al., 2004; Rop et al., 2018). Interestingly, some recent research suggests the effect of task-irrelevant information on learning efficiency is not necessarily detrimental; it may hamper basic learning processes, but it may also sustain them (Hazan-Liran & Miller, 2017; Miller et al., 2019).

To examine how competing task-irrelevant information creates an extraneous cognitive load interfering with learning efficiency, Hazan-Liran and Miller (2017) used a combination of the symbol-digit coding subtest (Wechsler Adult Intelligence Scale-III, 1997) and the Stroop color paradigm (Stroop, 1935) and asked university students to learn associations between eight base-code words (BCWs) that were either color names (e.g., blue, yellow) or color-related word concepts (e.g., sky, banana) and eight digits. The task was to apply this knowledge while rapidly completing rows of BCWs with their corresponding digits, as shown to participants in a permanently presented example section.

The paradigm, holding element interactivity constantly high, was implemented in three experimental conditions. In the first condition, the digits’ ink color overlapped with the core or peripheral semantics of the BCW stimuli (e.g., the digit related to ‘blue’ or ‘sky’ was presented in blue). In the second condition, the digits’ ink color was incongruent with the BCW stimuli’s semantics (e.g., the digit related to ‘blue’ or ‘sky’ was presented in red). In the third condition, the BCW stimuli and digits were all presented in achromatic black ink, a color assumed to provide a control to the two other conditions. Although in principle, digits’ ink color lacks relevance when learning an association between a BCW and a digit, it had a major impact on learning efficiency when it interacted with the BCWs’ color semantics. This impact was highly significant, even when task-irrelevant information referenced only peripheral properties of to-be-processed information (e.g., color-related word concepts). Of particular importance, the evidence strongly suggested task-irrelevant information had the potential to both enhance and undermine learning efficiency. In other words, its influence was detrimental when it conflicted with the semantics of the processed task-relevant information. In contrast, when its semantics overlapped those of to-be-processed information, it enhanced learning outcomes by strengthening the learned associations’ imprint in WM, hence facilitating their transfer to more permanent memory. As a consequence, the BCWs’ completion with their corresponding digits in the test section ceased to be dependent on a time-consuming lookup of particular associations in the example section. This gain was reflected in an increase in the number of word/digit completions achieved in a given time (two minutes).

The Hazan-Liran and Miller study (2017) convincingly demonstrated both inhibitory and facilitating effects on learning outcomes in the presence of task-irrelevant color information under well-controlled experimental conditions. To gain deeper insight into the origins of the inhibition, Miller et al. (2019), using the same basic paradigm with a new sample of university students, compared the impact of task-irrelevant color information on learning outcomes for color-related words (color names and color-related word concepts) and color-unrelated words (adjectives and nouns lacking color properties). As in the Hazan-Liran and Miller study, the task-irrelevant digit ink color markedly biased learning efficiency for color-related stimulus materials. However, its impact was strikingly absent when stimulus materials did not have color properties, suggesting the inhibition from task-irrelevant information, both positive and negative, was basically semantic in nature and could not be traced to a perceptual phenomenon.

In sum, both experiments suggested task-irrelevant information has the potential to modify the efficiency of learning processes contingent on whether or not it semantically interacts with the encoding of properties inherent to the information processed in the course of learning. Note, however, that in the above studies, the task-irrelevant color information was an inherent constituent of the to-be-learned information itself, namely, the digits’ ink color. It may therefore be argued that although in principle, it was irrelevant, in practice, participants couldn’t avoid processing it. The question is whether moving such task-irrelevant information from its central location on the digits to the background, a more external location, and increasing its salience further alters its impact on learning the associations between digits and color-related words. To answer this question, we conducted two experiments using a modified version of the Hazan-Liran and Miller (2017) paradigm. The original paradigm combines principles of the symbol-digit coding subtest (Wechsler Adult Intelligence Scale-III, 1997) and features of the classical Stroop paradigm (Stroop, 1935).

In these experiments, we sought to broaden our understanding of CLT by exploring the impact of task-irrelevant information (extraneous cognitive load) on learning efficiency and its semantic impact on learning outcomes. More specifically, we examined how the salience and location of irrelevant information – specifically, color information not directly related to the task at hand – can either facilitate or inhibit learning processes. We argued that the salience of irrelevant information may modify learning efficiency depending on its semantic overlap with to-be-learned material. We aimed to show that not all extraneous loads are detrimental; instead, their effects are nuanced and contingent on their interaction with the learners’ cognitive processing of relevant information. In other words, we sought to add to the literature by showing that the efficiency of information processing and learning can be influenced by seemingly irrelevant details, thus offering a more nuanced view on how cognitive load is managed. If not all extraneous information detracts from learning outcomes, and the impact of such information is contingent on its saliency and the context in which learning occurs, this suggests a more complex interaction within cognitive processes than previously understood and yields insights into optimizing instructional designs to enhance learning efficiency.

## Experiment 1: Impact of Task-Irrelevant Color Information Location and Salience on Learning Associations between Color Names and Digits

**Figure 1:**
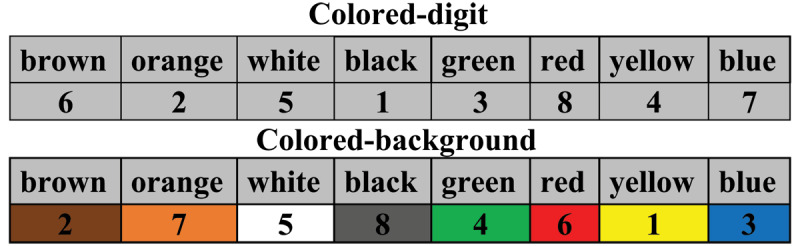
Variations in Task-Irrelevant Color Information Location and Salience (exemplified in English).

The aim of Experiment 1 was to clarify whether and how shifting the task-irrelevant ink color from the task-relevant digits to the background while simultaneously increasing its salience altered the efficiency of learning associations between digits and color names. For this purpose, we asked university students to rapidly complete rows of color names, which served as BCWs, with corresponding digits, as exemplified in a permanent example section. The experiment was administered under two distinct conditions. In the first, students performed the completion task with the task-irrelevant information (extraneous cognitive load) as an inherent property of the ink color of the BCWs’ corresponding digits (Figure 1). In the second condition, the task-irrelevant information was an inherent property of the digits’ background. Each condition comprised three sub-conditions: (1) the task-irrelevant digit ink or digit background color was congruent with the corresponding BCW’s semantics; (2) the task-irrelevant digit ink or digit background color was incongruent with the corresponding BCW’s semantics; (3) BCWs and their corresponding digits in the example section were both printed in black ink (control condition). Black is considered an achromatic control color and does not seem to interfere with the learning process (see Hazan-Liran & Miller, 2017; Miller et al., 2019).

A basic assumption in Experiment 1 was that task-irrelevant color information would bias the efficiency with which associations between color names and digits were learned. The magnitude of such bias was assumed to be contingent on the task-irrelevant information’s location and salience (digit ink color vs. digit background color), relative to the to-be-processed task-relevant information. Another assumption was that the nature of such bias was contingent on the degree to which the task-irrelevant information sustained (facilitation) or hampered (inhibition) this learning process. We tested the following specific hypotheses:

1. Task-irrelevant color information, if congruent with the color names’ semantics, will facilitate the learning of associations between color names and digits, contingent on this information’s location and salience. In other words, facilitation originating from task-irrelevant color information will be more pronounced in the colored-digit condition than in the colored-background condition, because in the former condition, color is an inherent property of the to-be-processed task-relevant information, i.e., the digits.
2. Task-irrelevant color information, if not congruent with the color names’ semantics, will inhibit the learning of associations between color names and digits, contingent on this information’s location and salience. In other words, learning inhibition rooted in task-irrelevant color information will be more pronounced in the colored-digit condition than in the colored-background condition.

### Experiment 1 Method

#### Participants

Sixty university students, 22 males and 38 females, age range 18–39 (M = 26.23), participated in Experiment 1. All were randomly sampled from different departments at the University of Haifa and were paid for participation. Only students without sensory impairments and learning disabilities were included in the study sample. Hebrew was the first spoken and read language of all participants.

Participants were randomly assigned to two experimental groups, 30 individuals per group. The first group was tested with the three sub-conditions of the *digit* condition and the second with those of the *background* condition.

#### Design and Stimuli

Experiment 1 was implemented as a paper and pencil task comprising an example section permanently visible to the participants and a test section provided on an A4 sheet (see Appendix A). The test section comprised several rows of BCWs with an empty field below each row, into which participants were asked to copy as fast as possible the corresponding digit, as shown in an example section located above the test section. To reveal the way task-irrelevant information impacts learning and the way it is modified by its location and salience, we developed six versions of the paradigm. In three of these versions, task-irrelevant information was introduced by manipulating the digits’ ink color in the example section, and in the remaining three, task-irrelevant information was introduced by manipulating the digits’ background color in the example section (see Figure 1).

**Figure 2:**
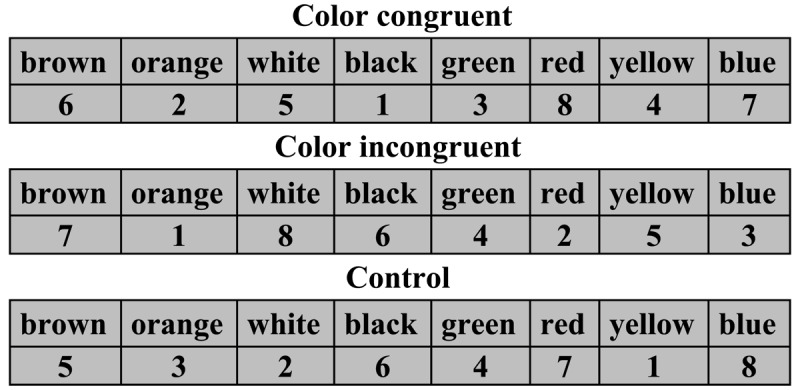
Three Sub-Conditions of the Colored-Digit Condition (exemplified in English).

Each of the two conditions had three sub-conditions: congruent, incongruent, and neutral (control). For the color-congruent sub-condition, in the example section, the digits’ ink color or digits’ background color was the same as the colors represented by the eight BCWs. For the color-incongruent sub-condition, the digits’ ink color or background color was different from the colors represented by the BCWs. In the control sub-condition, the color names and digits were all presented in black ink (see Figure 2). The assumption was that black – unlike other colors – is treated by the brain as neutral (achromatic). The sub-conditions of the colored-digit condition were identical to those of the colored-background condition, except that in the latter, the task-irrelevant color information was a property of the digits’ background, not their ink color.

The design of the test section below the example section in each of the sub-conditions was basically the same. It was comprised of 10 rows of rectangular fields, each containing one of the eight color names randomly set into it and an empty field below into which participants were asked to copy the corresponding digit (approximately 120 fields). The order of the BCWs in the example section and the test section was randomized across the different sub-conditions. The first four items of the top row of the test section were used for practice (see Appendix A).

#### Procedure

**Figure 3:**
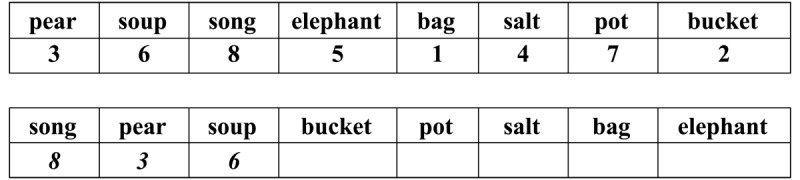
Example of BCWs and Corresponding Digits (both in black ink). *Note*: The words used here as examples were not used in the experiments. The three digits in italics represent the task exemplification.

After we received approval from the Institutional Ethics Committee for conducting human trials, and after participants had signed an informed consent form, participants were tested individually by a trained assistant in the authors’ lab. The order in which they performed the different sub-conditions rotated. To demonstrate the task requirements, the assistant used an example sheet (see Figure 3) with the following instructions:

Here you see eight Hebrew words presented side by side in rectangular fields (assistant points to example; see Figure 3). Below each word is a digit from 1 to 8. Beneath the example, you see another row of these words but this time in a different order and with an empty field below each word. The task is to learn which digit goes with which word and to complete each of the empty fields with its appropriate digit as exemplified.

The assistant then pointed to the four digits provided and ensured the participant’s understanding of the task requirements. After receiving confirmation, the assistant administered the three sub-conditions of Experiment 1, counterbalancing their order. To prevent carry-over effects of information held in WM, between each two conditions, the participants performed a computerized same/different task asking them to determine as fast as possible the identicalness of word dyads.

The participants were given the various experimental sheets (e.g., Appendix A). They initially saw only the example section and the first four BCWs of the test section; the remainder was covered by a blank piece of cardboard. So that we could reassess task understanding and also provide a warm-up, participants first completed the four visible items with their corresponding digits as exemplified in the example section. Participants’ learning efficiency in the test section was assessed only after practice indicated their understanding of the task.

Before actual testing began, the assistant told the participants that they would now be tested and instructed them:

When I remove the card board that covers the remainder of the sheet complete as many words as possible with their corresponding digits. Fill the digits in, word by word, from the right to the left (note: Hebrew is written from right to left), and progress row by row from the top to the bottom. Don’t skip words and try to avoid errors. If you make an error don’t correct it but move on, completing the remaining empty field with the appropriate digits. Please stop working immediately when I say ‘STOP’. Now put your pencil close to where you will start with the indication of the words’ corresponding digits. Are you ready?

The moment participants indicated their readiness, the assistant removed the coversheet and set a stopwatch. The participants were stopped after exactly two minutes. Their total number of correct and incorrect completions (the two dependent variables measured in the experiment) were recorded for further analysis. None of the participants managed to complete the whole test section. Note: the assistant did not inform the participants of the color manipulations introduced. Before being tested in the next condition, the participants performed the same/different categorization task.

### Publicly available data link

https://osf.io/3tvfb/?view_only=d6cfa9efe8ae4ded8d940e9d9df4662e.

### Experiment 1 Results

We analyzed the participant groups’ performance using a Two-way ANOVA with repeated measures mixed design procedure, computing task-irrelevant information’s location (digit, background) as a between-subject factor and color congruity (congruent, incongruent, control) as a within-subject factor. As error rates were very low (range .03 to 1.03), we did not analyze them separately. However, to account for their existence, we subtracted them from the participants’ overall number of digit completions when calculating their actual performance level. The average number of completions for the two color-location conditions and the three color-congruity sub-conditions, including their standard deviations, are presented in Table 1.

**Table 1:** Mean Performance for Color Names (standard deviations in parentheses).


CONGRUENCY	COLORED DIGITS	COLORED BACKGROUND

Color congruent	86.23 (16.47)	87.00 (13.70)

Color neutral	70.57 (12.87)	64.13 (9.40)

Color incongruent	57.60 (13.03)	47.47 (8.47)

Total	71.46 (12.25)	66.20 (19.47)


*Note*: Performance is the average number of correct completions minus error rates.

**Figure 4:**
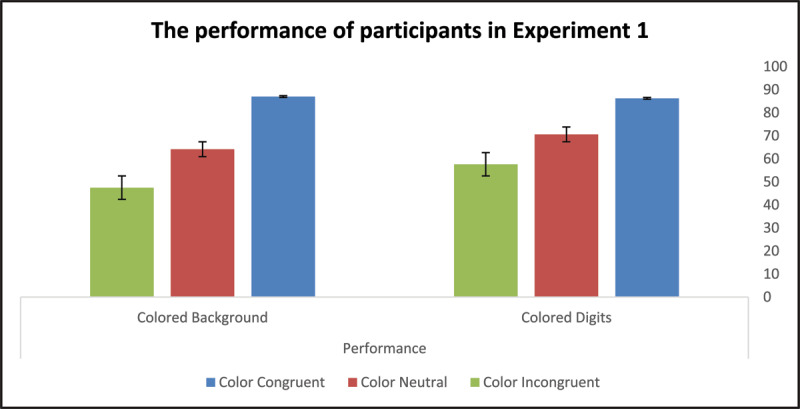
Participants’ Performance in Experiments 1.

**Figure 5:**
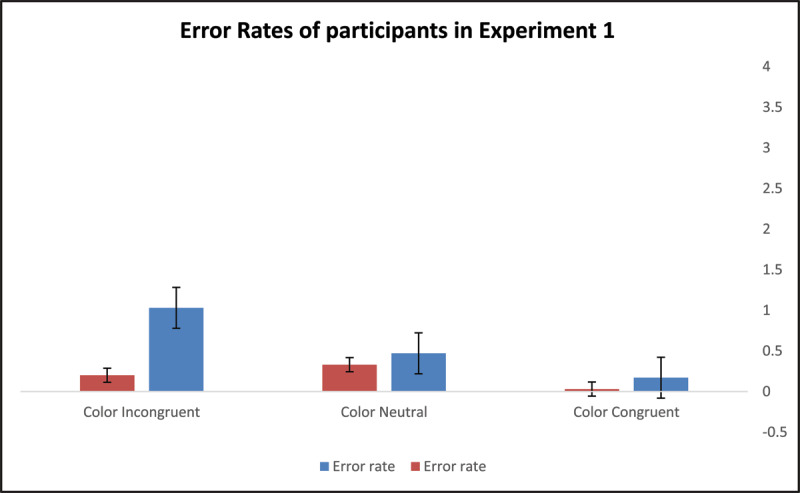
Participants’ error rates in Experiments 1.

The between-subject effect representing color location did not reach statistical significance, *F*(1,58) = 3.84, *p* = .055, *η*^2^*_p_* = .06. This seems to suggest that, overall, the efficiency with which participants learned associations between color names and digits was not affected by the location of the task-irrelevant color information. The effect of color congruity was statistically significant, *F*(2,58) = 145.86, *p* < .001, *η*^2^*_p_* = .72, indicating that the number of completions in two minutes was strongly biased by the congruity of the task-irrelevant color information with the color names’ semantics. There was a significant interaction between the color-location effect and the color-congruity effect *F*(2,58) = 5.09, *p* = .028, *η*^2^*_p_* = .08, implying that color location further modified the impact of the color-congruity effects on learning. Figures 4 and 5 illustrate the performance and error rates of participants in Experiment 1 under the three possible experimental conditions.

We conducted a series of post-hoc analyses to clarify how the manipulation of color location influenced facilitation and inhibition effects (color location × color congruity interaction) originating from the task-irrelevant color information. Conducting this analysis was crucial, as the comparison of the two color-neutral conditions (all black ink) indicated that the two participant groups statistically differed in their default learning skills, *F*(1,58) = 4.89, *p* = .031. In other words, participants who learned the association between BCWs and digits in the colored-digit condition made more completions in the control sub-condition than participants who learned the same associations in the colored-background condition.

To disclose the final significance of the color location × color congruity interaction, we created two differential scores, one representing the facilitation effect and the other representing the inhibition effect. The facilitation effect (for colored digits, M = 15.67, SD = 12.74; for colored background, M = 22.87, SD = 12.74) was calculated by subtracting completion rates in the control condition from completion rates in the color-congruent condition. The inhibition effect (for colored digits, M = –12.97, SD = 10.17; for colored background, M = –16.67, SD = 8.45) was calculated by subtracting completion rates in the color-incongruent condition from completion rates in the color-control condition (see Table 1). To elucidate whether color location differently biased color-congruency effects, we ran a Two-way ANOVA with repeated measures mixed design procedure, with color location (digit, background) as a between-subject factor and color congruity (facilitation effect, inhibition effect) as a within-subject factor. A statistically significant interaction between the two main effects, *F*(1,58) = 5.09, *p* = .028, *η*^2^*_p_* = .08, suggested color location biased the size of the two color-congruity effects differently.

To determine the final significance of this interaction we conducted two One-way ANOVAs considering how color location modified the color-congruity-related facilitation and inhibition effects. Results indicated that the accentuated colored-background condition significantly increased the facilitation effect originating from color congruity, *F*(1,59) = 5.09, *p* = .028, but did not noticeably modify the size of the inhibition effect resulting from color incongruity, *F*(1,59) = 2.35, *p* = .131.

### Experiment 1 Discussion

CLT predicts that the presence of task-irrelevant information interferes with learning processes (Sweller, 2010; Sweller et al., 2011; Sweller & Chandler, 1994; van Merriënboer & Ayres, 2005), especially when the intrinsic cognitive load is high (Lavie et al., 2004; Rop et al., 2018). In a series of experiments, Hazan-Liran and Miller (2017) and Miller et al. (2019) further demonstrated that such inhibition is essentially semantic and has the potential to both enhance and inhibit learning efficiency. The question is whether relocating and accentuating task-irrelevant information from the focus of to-be-learned materials further modifies the impact of an extraneous cognitive load. Experiment 1 was designed to answer this question by testing learning efficiency with two modified versions of the original Hazan-Liran and Miller paradigm (2017). In one of these versions, the task-irrelevant color was an immanent component of the digits (colored-digit); i.e., it was a component the learner’s eyes inevitably focused on when learning the association between digits and BCWs. In the other version, the task-irrelevant color information was assigned to the digits’ surrounding background; i.e., it was relocated and accentuated relative to the immediate focus of the to-be-processed information.

Building on Miller and Hazan-Liran’s previous findings, we hypothesized that task-irrelevant color information, if congruent with the color names’ semantics, would facilitate the learning of associations between color names and digits in both experimental conditions (colored-digit, colored-background). In the same vein, we hypothesized that task-irrelevant color information, if not congruent with the color names’ semantics, would markedly inhibit the learning of associations between color names and digits in both experimental conditions. Findings fully corroborated both hypotheses, implying that relocating and accentuating task-irrelevant color information from being immanent to the processed stimulus to a stimulus-external location did not result in the avoidance of its processing, nor did it essentially alter the way it biased learning efficiency. This suggests that when learning under conditions of high element interactivity, learners may lack cognitive resources capable of stopping task-irrelevant information from influencing the learning process.

We also hypothesized that facilitation and inhibition effects originating from task-irrelevant color information would be more pronounced in the colored-digit condition than the colored-background condition. As an imminent, although task-irrelevant, property of the stimuli in the former case, color is unavoidably in the center of perceptual processes involved in the identification of the digits themselves and can hardly be ignored. In the latter case, color is not directly linked to the digit stimuli and therefore should logically have a less influential impact on the learner’s learning efficiency. Findings from Experiment 1 ran counter to these two hypotheses. Contrary to expectations, the learning facilitation effect in the color-congruent condition was significantly more pronounced when task-irrelevant color information was a quality of the digits’ surrounding background than when it was implemented as an immanent property of digits’ ink color. Moreover, the learning inhibition effect originating from color incongruity was not significantly more pronounced in the colored-digit condition than in the colored-background condition.

Both findings are puzzling, as they seem to suggest that the task-irrelevant color information’s location *per se* was not a key factor in its impact on learning efficiency. Going on intuition, it may, of course, be argued that the accentuated background color explains the deviance of our findings, not the digits’ ink color. However, this explanation seems untenable, as it assumes increased background color salience should have influenced the magnitude of facilitation and inhibition effects in a similar way, not asymmetrically as was actually the case. In other words, in instances when the semantics of task-irrelevant background color overlapped with the semantics of the color names (congruent condition), color salience enhanced learning efficiency beyond the enhancement found in the colored-digit condition. Meanwhile, in instances when the task-irrelevant background color conflicted with the semantics of the color names (incongruent condition), a different processing scenario seemed to attenuate the accentuated background colors’ potential to additionally harm learning processes. An attempt to explain the essence of this apparent asymmetry appears in the General Discussion; in the proposed explanation, we directly refer to the perceptual/cognitive processing scenarios assumed to underlie the learning of associations between BCWs and digits.

In sum, evidence from Experiment 1 implies that when dealing with learning tasks with inherently high element interactivity, learners are unable to block perceived task-irrelevant information from entering the learning process (e.g., Sweller et al., 2011). Such information, contingent on the nature of its semantic overlap with to-be processed information, tends to either sustain or hamper learning efficiency. Its relocation from the focus of to-be-learned information does not seem to further influence learning efficiency *per se*. However, the findings also suggest that in interaction with task-irrelevant information’s increased salience in the background condition, background color salience enhances learning efficiency in instances when its semantics overlap with those of the to-be-processed information (the color names), but not when there is no such overlap.

## Experiment 2: Impact of Task-Irrelevant Color Information Location and Salience on Learning Associations between Color-Related Word Concepts and Digits

In line with previous research (Hazan-Liran & Miller, 2017; Miller et al., 2019) and our proposed hypotheses, evidence from Experiment 1 suggested the presence of task-irrelevant information modifies learning efficiency, depending on the degree of its overlap with the semantics of the to-be-learned information. However, the relocation of task-irrelevant color information from the immediate focus of information processing to the background did not, as hypothesized, moderate its impact on learning efficiency. On the contrary, possibly because of its increased salience in the background condition, color actually seemed to enhance learning efficiency when its semantics were congruent with those of the to-be-processed information, yet counterintuitively, learning was not hampered when they were incongruent. Accordingly, we wondered whether the effects of the salience of the digits’ background color on learning efficiency we found in Experiment 1 could generalize to learning contexts in which the potential of task-irrelevant color information to modify learning outcomes seemed less straightforward.

To answer this question, we tested new samples of university students with a new-code learning paradigm; this was identical to the one used in Experiment 1, except that it used color-related word concepts (sky) instead of color names (blue) as stimulus materials. In learning associations between such stimulus words and digits, facilitation and inhibition effects originating from the presence of task-irrelevant color information have been found to be essentially similar, although somewhat more moderated than those for color names (Hazan-Liran & Miller, 2017). Note: as with color-related word concepts, effects from task-irrelevant color information result from this information’s interaction with the stimuli’s attributes, not with their core meaning. Therefore, if relocating the task-irrelevant color information from the digits to their background was found to bias participants’ learning efficiency, as in Experiment 1, effects produced by the salience of task-irrelevant information should be accounted for as an additional intrinsic component in explaining variance in the efficiency of learning processes.

With direct reference to how task-irrelevant color information was found to influence learning of associations between color names and digits in Experiment 1, we developed three new research hypotheses and tested them in Experiment 2.

1. Task-irrelevant color information, contingent on its congruency with the color-related word concepts’ semantics, will either facilitate or hamper the learning of associations between color-related word concepts and digits.
2. Facilitation originating from task-irrelevant color information will be more pronounced in the colored-background condition (strong color salience) than in the colored-digit condition (weak color salience).
3. Inhibition originating from task-irrelevant color information will be similar in the colored-background condition and the colored-digit condition.

### Experiment 2 Method

#### Participants

A new study sample of 60 university students was recruited based on the same criteria as Experiment 1 and randomly divided into two equal groups, one tested in the colored-digit condition and the other in the colored-background condition.

#### Design, Stimuli, and Procedure

The methodology of Experiment 2 was identical to that of Experiment 1, except that stimulus materials were not color names but color-related word concepts: sky (blue), banana (yellow), strawberry (red), grass (green), darkness (black), whipped cream (white), orange (orange), mud (brown). A detailed description of the color-related word concepts is provided in Appendix B.

### Experiment 2 Results

None of the participants managed to complete the whole test section. As in Experiment 1, error rates in Experiment 2 were very low (range .10 to .33) and were therefore not analyzed independently. Table 2 displays mean performance for the two color-location conditions (digit, background) and the three color-congruity sub-conditions (congruent, control, incongruent), including their standard deviations.

**Table 2:** Mean Performance for Color-Related Word Concepts (standard deviations in parentheses).


CONGRUENCY	COLORED DIGITS	COLORED BACKGROUND

Color congruent	75.63 (14.99)	92.03 (16.77)

Color neutral	70.37 (14.47)	75.47 (12.33)

Color incongruent	63.70 (12.44)	64.80 (13.29)

Total	69.90 (11.66)	77.43 (18.04)


*Note*: Performance is the average number of correct completions minus error rates.

We used a Two-way ANOVA with repeated measures mixed design procedure, computing task-irrelevant information’s location as a between-subject factor and color congruity as a within-subject factor, to analyze the participant groups’ performance. The location main effect (colored-digit vs. colored-background) was statistically significant, *F*(1,58) = 6.30, *p* = .015, *η*^2^*_p_* = .10, indicating that, overall, participants who learned associations between BCWs and digits in the colored-digit condition underperformed participants who learned such associations in the colored-background condition. The color-congruity effect was statistically significant, *F*(2,58) = 30.94, *p* < .001, *η*^2^*_p_* = .35, implying that the congruity of the task-irrelevant color information with the color-related word concepts’ semantics altered the number of completions participants made in two minutes. The color-location effect was found to interact with the color-congruity effect, *F*(1,58) = 8.29, *p* = .006, *η*^2^*_p_* = .13, suggesting that moving the task-irrelevant color from the digit to the background further modified the way color congruity biased the participants’ learning efficiency.

**Figure 6:**
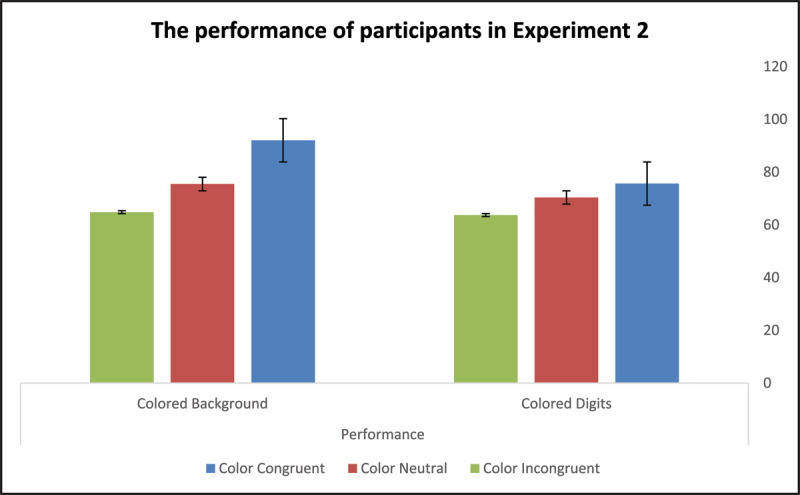
Participants’ Performance in Experiment 2

**Figure 7:**
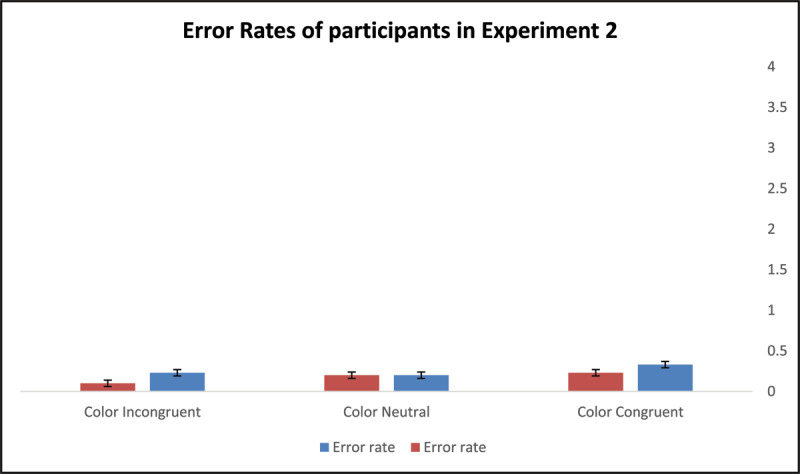
Participants’ error rates in Experiment 2.

Note: unlike Experiment 1, in Experiment 2, comparing the participant groups on the color-neutral condition failed to reveal statistically significant differences with respect to their default learning skills, *F*(1,59) = 2.16, *p* = .147. As in Experiment 1, to yield a more profound understanding of how task-irrelevant color’s location biased the participants’ performance, we calculated two scores; one reflecting facilitation and the other inhibition effects with reference to performance in the control condition (see Table 2). We conducted two lines of post-hoc analyses to clarify whether and how color location noticeably modified the size of facilitation (for colored digits, M = 5.26, SD = 14.50; for colored background, M = 16.57.87, SD = 15.88) and inhibition effects (for colored digits, M = –6.67, SD = 9.78; for colored background, M = –10.67, SD = 9.62). The first analysis, implemented by means of a Two-way ANOVA with repeated measures mixed design procedure, computed color location (digit, background) as a between-subject factor and color congruity (facilitation effect, inhibition effect) as a within-subject factor. A statistically significant interaction between the two main effects, *F*(1,58) = 13.67, *p* < .001, *η*^2^*_p_* = .19, indicated that the way color location modified facilitation and inhibition effects was not uniform. To determine the final nature of this variance, we ran two One-way ANOVAs, one examining how color location biased color-congruity-related facilitation and the second examining color-incongruity-related inhibition. Results suggested that the colored-background condition significantly increased the facilitation effect originating from color congruity, *F*(1,59) = 8.29, *p* = .006, but did not noticeably modify the size of the inhibition effect resulting from color incongruity, *F*(1,59) = 2.55, *p* = .116. Figures 6 and 7 illustrate the performance and error rates of participants in Experiment 2 under the three possible experimental conditions.

Experiment 2’s hypotheses predicted that task-irrelevant color information would interact with the color attributes of color-related word concepts. More specifically, we hypothesized that depending on the nature of this color information’s congruency with these color attributes, learning efficiency would either be facilitated or hampered. The evidence fully substantiated this hypothesis. Task-irrelevant digit color enhanced learning efficiency in instances when the word concepts’ color attributes were congruent with the digits’ color and reduced it in instances when they were incongruent. This performance pattern converges with conclusions drawn from Experiment 1 and previous research (Hazan-Liran & Miller, 2017; Miller et al., 2019) to suggest that learners struggle to control bias from task-irrelevant information while learning.

In Experiment 1, we predicted that assigning a task-irrelevant color to digits (colored-digit condition) would affect learning efficiency more than inserting it as a digit background property (background-color condition). In the former case, the task-irrelevant color is an important part of the information learners have to process in order to learn the association between color names and digits. However, we found that allocating task-irrelevant color information to the digits’ background led to enhanced learning in instances when this color overlapped with the semantics of the color names, but not when it was incongruent. Taken together, these findings suggest the impact on learning of increased salience of task-irrelevant information is contingent on how this information interacts with task-relevant information. Experiment 2 used color-related word concepts instead of color names to corroborate this primary conclusion, testing three research hypotheses reformulated in line with Experiment 1’s findings.

The second of these hypotheses predicted that facilitation originating from task-irrelevant color information would be more pronounced in the colored-background condition (strong color salience) than in the colored-digit condition (weak color salience). The third predicted that in the colored-background condition, the inhibition effect on learning originating from task-irrelevant color information would be similar to that in the colored-digit condition. The experiment’s findings fully substantiated both hypotheses. Supporting insights gained from Experiment 1, the findings imply that learning associations between BCWs and digits initiates a processing scenario that gains from the salience of task-irrelevant color information selectively when it has the potential to enhance basic learning. A different processing scenario, however, seems to moderate the potentially negative effect on basic learning of accentuating task-irrelevant color information. The fact that such apparently different processing scenarios were indicated from the thorough examination of the findings of the different color name conditions (color as a core meaning), as well as the color-related word concept conditions (color simply as an attribute), and were found in different participant samples, is noteworthy. As a matter of fact, it corroborates the conjecture that the effects of increased salience of task-irrelevant information on learning are a byproduct of the quality of this information’s semantic interaction with the to-be-processed task-relevant information.

## General Discussion

Evidence from Experiments 1 and 2 indicates that when task-irrelevant information penetrates the learning process, an extraneous cognitive load is created, impacting the learner’s efficiency in learning task-relevant information. It also shows that certain properties of task-irrelevant information – in our experiments, the enhanced salience of the background colors – further modify the way extraneous cognitive load biases learning efficiency. The question is: what processing scenarios explain the occurrence of such diverse impacts on learning efficiency? Answering this question is crucial, as it has the potential to extend CLT’s understanding of the nature of the factors underlying variance in learning efficiency.

Finding an answer first requires understanding the basic processing scenario underlying the learning of the associations between BCWs and digits, a task required in both experiments. We begin by discussing the scenario in relation to the control sub-conditions of both experiments, i.e., without the presence of task-irrelevant color information. BCWs and digits in these instances were both presented in achromatic black ink (see Appendix), a color that does not seem to produce extraneous cognitive load (e.g., Hazan-Liran & Miller, 2017; Miller et al., 2019). In this default scenario, completing BCWs with their corresponding digits in the test section (see Appendix A) is likely to have involved the following processing steps: (1) recognition of a presently targeted BCW in the test section, based on the learner’s permanent phonological and/or orthographic lexicon; (2) transfer of a phonological and/or orthographic representation into WM and its rehearsal to sustain its temporary retention; this operation may also trigger the retrieval of the BCW’s semantics, i.e., its color or color attribute; (3) relocation of attention from the test section to the eight BCW candidates in the example section in search of a match; (4) with the finding of a match, the assignment of attention to the digit below the matching BCW candidate for its recognition and subsequent entry into WM; (5) subvocal rehearsal of the BCW and its corresponding digit in WM, while relocating attention back to the test section to assign the digit below the targeted BCW; (6) repetition of the procedure for subsequently targeted BCWs.

In the above scenario, learning an association between a BCW and a digit is intrinsically linked with the subsequent simultaneous rehearsal of their association from Step 5 on. Once the association is internalized, the learner does not have to look up already learned associations in the example section in a time-consuming process, a gain that increases the BCW/digit completion rate within a given timespan. Of special note, however, the addition of task-irrelevant color information (extraneous cognitive load), whether to the digits themselves or to their surrounding background, seems to essentially modify the processing scenario underlying the learning of associations between BCWs and digits. This holds true whether such associations are learned under color-congruent or color-incongruent conditions.

We now delineate the assumed modifications in the processing scenario where the digits’ ink or background color was congruent with the targeted BCWs’ color semantics. As in the control conditions, in the color-congruent conditions, the learner, prior to the internalization of a specific BCW/digit association, needs to refer to the eight candidates in the example section in the search for a match (Step 3) and do so while holding the targeted BCW’s phonology, orthography, and semantics activated in WM. There are at least two potential scenarios that seem to explain the enhanced efficiency we found in learning associations in this condition. The first reflects a lexico-semantic strategy where the learner recognizes the targeted BCW’s match, similar to the control condition, by successively processing the eight candidates in the example section phonologically and/or orthographically until there is a hit. With this hit – due to the processing of the digits’ task-irrelevant ink or background color – the already activated representation of the targeted BCW in WM becomes semantically and lexically double-coded. Such double coding solidifies the BCW’s representation and frees attention resources which, when redirected to intensify rehearsal processes, accelerate the consolidation of BCW/digit associations; the result is an increased base-code/digit completion rate, relative to the control condition.

The second scenario reflects a primarily semantic processing strategy, whereby after relocating attention from the test section to the example section (Step 3), the learner’s attention, because of the targeted BCW’s activated semantics, becomes instantly attracted by the digits’ ink or background color, rather than being directed to the scanning of the BCW candidates in the example section in search of a match (Step 4). Because of its physical closeness to the digit, the processing of the task-irrelevant color is likely to lead to the retrieval of the digit’s semantics but, at the same time, the imprint of the targeted BCW’s already activated representation in WM will be solidified. This, in turn, will prime the recognition of the digit’s parallel BCW candidate. Together, as in the first scenario, these latter operations free attention resources, and this intensifies rehearsal processes; this, in turn, accelerates the internalization of the association between the BCW and the digit, ultimately resulting in an increase in the base-code/digit completion rate, relative to that in the control condition.

Both scenarios reasonably explain why, in comparison to the control condition, in the congruent conditions, participants manifested enhanced learning, reflected in a higher BCW/digit completion rate. However, there is an essential difference between the two. Directing attention to the targeted BCW candidate is a more salient feature of the second scenario than the first. This scenario skips the time-consuming processing of BCW candidates until the target candidate is recognized. It is also reasonable to assume that the increased salience of the task-irrelevant color information in the background sub-condition not only attracts the learner’s attention faster, but also more strongly supports the targeted word’s representation in WM. If this is indeed the case, the observation that learning advantages in this sub-condition were even more pronounced than in the colored-digit sub-condition makes sense. However, are the two scenarios adequate to explain the participants’ learning efficiency in instances when the task-irrelevant ink or background color information was incongruent with the color semantics of the BCW candidates? Answering this question is important, as in this sub-condition, increased salience of the task-irrelevant background color did not have a statistically significant additive negative effect on our participants’ learning efficiency.

As discussed for the color-congruent condition, after learners shift attention from the test section to the example section looking for the targeted BCW’s candidate, their attention is likely to be first attracted by the digit ink or background color that corresponds to the color semantics of the BCW stored in WM. The same is probably true for learning the association between BCWs and digits in the color-incongruent condition. However, unlike the processing scenario assumed to occur in the color-congruent condition, in this particular condition, the recognition of the incongruity between the in-WM-retained representation of the targeted BCW and the digit’s corresponding BCW candidate forces the learner to switch to the lexico-semantic processing strategy. In other words, the search for a targeted BCW match proceeds by lexically processing the BCW candidates in the example section and comparing them to the in-WM-activated lexical representation of the targeted BCW. This change from a rapid primarily semantic search strategy to a slower lexico-semantic search strategy is likely to require the prolonged retention of the targeted BCW’s imprint in WM while counteracting its gradual fading via additional subvocal rehearsal. Yet not only is this lexico-semantic search process more time-consuming, but in the presence of misleading digit ink or background color, the learner is also forced to recruit attention resources to counteract the processing of the task-irrelevant color to prevent failure in learning the association between BCWs and digits following a hit. Moreover, unlike in the color-congruent condition, in the color-incongruent condition, the digit’s ink or background color neither double-codes the targeted BCW’s imprint in WM nor primes the recognition of the targeted BCW candidate.

When all these processing constraints are considered together, the reduced learning efficiency indicative of our participants’ performance in the color-incongruent conditions, reflected in a markedly reduced BCW/digit completion rate relative to the color-neutral control and color-congruent conditions, makes sense. Moreover, in completing the color-incongruent conditions, participants most likely made a substantial effort to block the processing of the misleading color information; therefore, the finding that, unlike in the color-congruent condition, their performance in the colored-background conditions was not particularly harmed by increase color salience, makes sense as well.

In summary, findings from this study and evidence reported elsewhere (e.g., Hazan-Liran & Miller, 2017; Miller et al., 2019) yield some important insights. First, they suggest extraneous cognitive load, although principally irrelevant for completing a learning task, has the potential to either sustain or undermine learning processes by varying the overall cognitive load. Second, the study’s scientific basis allows the cognitive mechanisms underlying the positive and negative impact of extraneous cognitive load on basic learning in the presence of high element interactivity to be characterized. In particular, the findings suggest that apparently task-irrelevant information can be transformed into a means that helps learners pay attention to task-relevant information by double-coding and/or priming parts of its recognition or retention in WM and by so doing, reduce overall cognitive load. Moreover, the more salient the processed task-irrelevant information, the stronger its contribution to learning efficiency seems to be. In the opposite case, task-irrelevant information becomes an extraneous cognitive load that binds attention resources to suppress this information’s task-distracting potential, consequently reducing the amount of attention learners can recruit for the processing of to-be-learned information and resulting in reduced learning efficiency. Findings actually seem to indicate that reduced learning in this case is a byproduct of sacrificing cognitive resources to keep task-irrelevant information from being processed rather than the result of its processing.

### Limitations and Future Research

Admittedly, the work has some limitations. The processing scenarios assumed to underlie the nature of extraneous cognitive load and their impact on learning efficiency are based on a primarily deductive logical interpretation of the findings obtained from a limited number of studies, all using variations of a single basic research paradigm. To corroborate our findings, future work should use methods that allow tracking the impact of extraneous cognitive load on learning in a straightforward manner. Such research should expand the investigation to more complex learning contexts to substantiate the generalizability of this study’s conclusions. The repeated finding that contrary to expectation, the increased color saliency of the colored-background condition modified only the facilitation effect, not the inhibition effect, came as a surprise. To substantiate conclusions drawn from the discussion of this disparity based on proposed processing scenarios, in future research, the learning paradigm we used should be implemented with additional saliency conditions, such as adding a small task-irrelevant patch of color near the digits or coloring the bottom of the squares comprising the digits in the example section.

## Data Accessibility Statement

The materials are available at https://drive.google.com/file/d/1wDYTiTbkaaqepAJ9YwvAY067_1S1DZ43/view?usp=drive_link. The link contains a Handbook that explains the names of the variables.

## Additional Files

The additional files for this article can be found as follows:

10.5334/joc.361.s1 Appendix A. Control Test Sheet (English translation of stimuli in parentheses).

10.5334/joc.361.s2 Appendix B. Mean Letter Length, Syllabic Length, and Word Frequency of Experimental Stimuli.
