# Supplementary material for: The Influence of Manipulating and Accentuating Task-Irrelevant Information on Learning Efficiency: Insights for Cognitive Load Theory
Source: J Cogn. 2024 Apr 18;7(1):36. doi: 10.5334/joc.361 (PMC11025566; doi:10.5334/joc.361)
Supplement: Appendix A. — Control Test Sheet (English translation of stimuli in parentheses). [file joc-7-1-361-s1.pdf]

Appendix A

Control Test Sheet (English translation of stimuli in parentheses)

(example) דוגמה

חום (brown)	כתום (orange)	לבן (white)	שחור (black)	ירוק (green)	אדום (red)	צהוב (yellow)	כחול (blue)
5	3	2	6	4	7	1	8

שחור	ירוק	צהוב	חום	כחול	כתום	לבן	אדום	כחול	חום	אדום	כחול

לבן	אדום	כחול	כתום	אדום	לבן	כתום	כחול	חום	צהוב	שחור	ירוק

צהוב	כתום	שחור	ירוק	כתום	כחול	אדום	לבן	ירוק	שחור	חום	צהוב

ירוק	כחול	אדום	שחור	צהוב	כחול	לבן	ירוק	חום	כתום	אדום	חום

צהוב	כחול	לבן	ירוק	חום	כתום	אדום	שחור	חום	לבן	צהוב	כתום

לבן	אדום	כחול	כתום	חום	צהוב	שחור	כתום	כחול	אדום	לבן	שחור

אדום	לבן	כתום	כחול	חום	צהוב	ירוק	שחור	ירוק	שחור	חום	צהוב

חום	צהוב	ירוק	אדום	שחור	ירוק	צהוב	חום	כחול	כתום	אדום	לבן

כחול	אדום	לבן	ירוק	שחור	חום	צהוב	לבן	אדום	כחול	כתום	שחור

שחור	צהוב	כחול	לבן	ירוק	חום	כתום	אדום	חום	צהוב	ירוק	שחור
