# Supplementary material for: The Influence of Manipulating and Accentuating Task-Irrelevant Information on Learning Efficiency: Insights for Cognitive Load Theory
Source: J Cogn. 2024 Apr 18;7(1):36. doi: 10.5334/joc.361 (PMC11025566; doi:10.5334/joc.361)
Supplement: Appendix B. — Mean Letter Length, Syllabic Length, and Word Frequency of Experimental Stimuli. [file joc-7-1-361-s2.pdf]

Appendix B

Mean Letter Length, Syllabic Length, and Word Frequency of Experimental Stimuli

Experimental Stimuli	Translation of Stimuli	Phonetic Transcription	Letter Length	Syllabic Length	Word Frequency
The eight color names					
חום	Brown	xum	3	1	47
כתום	Orange	katom	4	2	10
לבן	White	lavan	3	2	140
שחור	Black	Šaxor	4	2	76
ירוק	Green	yarok	4	2	46
אדום	Red	adom	4	2	66
צהוב	Yellow	ca'ov	4	2	30
כחול	Blue	kaxol	4	2	34
Means			3.75	1.87	56.12
The eight color-related word concepts					
שמיים	Sky	Šamaym	5	3	6
בננה	Banana	banana	4	3	9
תות	Strawberry	tut	3	1	6
דשא	Grass	deŠe	3	2	8
חושך	Darkness	xoŠex	4	2	11
קצפת	Whipped cream	kacefet	4	3	4
תפוז	Orange	tapuz	4	2	12
בוץ	Mud	boc	3	1	9
Means			3.75	2.12	8.12
